# Toward an explanation of cultural differences in subjective well-being: the role of positive emotion norms and positive illusions

**DOI:** 10.3389/fpsyg.2024.1356172

**Published:** 2024-06-20

**Authors:** Hyunji Kim, Joni Y. Sasaki

**Affiliations:** ^1^Institute of Confucian Philosophy and Culture, Sungkyunkwan University, Seoul, Republic of Korea; ^2^Department of Psychology, University of Hawaiʻi at Mānoa, Honolulu, HI, United States

**Keywords:** emotion norms, positive emotions, positive illusions, self-enhancement, halo bias, social cognition, well-being, culture

## Abstract

The present research explores the role of positive emotion norms and positive illusions in explaining the higher subjective well-being observed among Europeans compared to East Asians in Canada. Specifically, we investigate the underlying psychological mechanisms contributing to the prevalence of positive self-views among individuals with European backgrounds, characterized by individualism, versus those with East Asian backgrounds, associated with collectivism. Our study compares Europeans and East Asians in Canada to determine whether cultural norms regarding positive emotions account for the elevated positive self-views and subjective well-being in Europeans. With a sample of 225 participants (112 Europeans and 113 East Asians), our findings reveal significant indirect effects of culture on subjective well-being through positive emotion norms and positive illusions. This study highlights that Europeans, compared to East Asians, believe it is more appropriate to experience and express positive emotions, and this norm influences their positive self-views, subsequently impacting subjective well-being. These findings offer valuable insights into how cultural factors shape subjective well-being across different groups.

## Introduction

Psychological research consistently shows that people from individualistic cultures report greater life satisfaction than those from collectivistic cultures. This distinction in subjective well-being has often been attributed to the higher socio-economic status, such as income, of people in North America (Veenhoven, 1991; Diener et al., 1995, 1999; Ahuvia, 2002; Tan et al., 2020). Studies comparing nations and different regions within countries have identified factors like income disparities, gender equality, education, and healthcare systems as key to understanding the relatively higher subjective well-being of North Americans. These elements enable individuals to engage in pursuits that significantly boost their sense of well-being. Beyond economic and social factors, the phenomenon of positive illusions has been proposed as a reason for the higher subjective well-being observed among Europeans compared to Asians, with Europeans tending to hold a more optimistic view of themselves (Kim et al., 2012, 2016).

However, the psychological underpinnings of why Europeans, compared to East Asians, exhibit more positive self-views and higher subjective well-being remain underexplored. This study investigates a potential psychological factor: the cultural norm surrounding the experience and expression of positive emotions. While having good education, healthcare, and high income are important for individuals’ well-being, a crucial factor shaping one’s well-being may be beliefs about what is appropriate to feel in the cultural context where they live. That is, culture shapes individuals’ beliefs and thoughts on appropriateness of emotions, which in turn, influence their satisfaction with different domains in life – such as family relationship, friendship and financial situation, −and with life in general. Importantly, cultural norms of positive emotions may explain why people view themselves overly positively. By examining these aspects, the study aims to uncover why individuals of European background generally display more positive self-views and subjective well-being.

### Research on positive illusions

Positive illusions involve individuals’ tendencies to maintain positive self-images, bolster self-esteem, and cultivate positive emotions (Taylor and Brown, 1988). According to studies and theories on positive illusions, individuals are motivated to perceive their attributes and behaviors in an excessively positive light, such as overestimating their driving skills or believing they are well-liked by friends (Festinger, 1954; Svenson, 1981; Taylor and Brown, 1988). This inclination toward positive self-views extends to different domains–such as personal characteristics and behaviors, and perceptions of close others as well (Murray et al., 1996; Kwan et al., 2004; Wood et al., 2010).

One way to measure positive illusions is by using a structural equation model of personality traits called halo bias. Halo bias has been validated and replicated in previous research with other positive illusion measures. It reflects the tendency to evaluate one’s Big Five personality traits overly positively and has been associated with greater subjective well-being (Anusic et al., 2009; Kim et al., 2012, 2016). Comparative research has explored how individuals with a European background in North America assess themselves and their life satisfaction in contrast to those with an Asian background, both within North America and Asia. Previous research has found that European Canadians are more likely to view their own and their close friends’ personalities in an excessively positive light compared to Asian Canadians, a factor that contributes to their higher levels of life satisfaction (Kim et al., 2012). In other words, Europeans’ higher life satisfaction was explained by their positive self-views. While previous studies have suggested the *prioritization of positive emotions* (rather than an accurate perception of reality) by Europeans as a reason for their positive self-views, this hypothesis was not directly tested.

The present study aims to bridge the gap by introducing a psychological mediator—*the cultural norm of positive emotions*—as a potential explanation for the heightened positive illusions among Europeans and their subsequent influence on subjective well-being.

### Cultural norms of positive emotions as the psychological mediator

Cultural differences in self-perception and subjective well-being may be influenced by normative views on experiencing and displaying positive emotions. The relationship between emotional experiences and overall subjective well-being is well-documented across numerous studies. Research indicates that experiencing positive emotions is a critical factor in assessing well-being (Suh et al., 1998; Schimmack et al., 2005; Schwarz and Clore, 2007). In both individualistic and collectivistic cultures, positive emotions are positively correlated with individuals’ life satisfaction, with a stronger effect observed in individualistic cultures (e.g., Suh et al., 1998). The affect-as-information hypothesis (Schwarz and Clore, 1983) further supports this, suggesting that emotional experiences significantly influence judgments of life satisfaction, thereby highlighting how individuals incorporate their emotional experiences into their evaluations of life satisfaction. Additionally, positive emotions seem to hold greater significance for the subjective well-being of European North Americans compared to East Asians, indicating that cultural values surrounding positive emotions may influence the cultural norms and beliefs regarding positivity in different societies.

There is substantial evidence indicating that culture plays a significant role in shaping individuals’ perceptions of the appropriateness of experiencing and expressing positive emotions (Eid and Diener, 2001; Safdar et al., 2009; Sims et al., 2015). Research by Eid and Diener (2001) demonstrated that a majority of individuals from individualistic cultures, such as the United States and Australia, considered positive emotions more appropriate (with 83% of the sample in each country holding this view) compared to their counterparts in collectivistic cultures like China and Taiwan. This highlights the impact of cultural frameworks on the acceptance and expression of positive emotions.

People from different cultures also differ in the emotions they would like to feel. North Americans compared to East Asians said they would like to feel high arousal positive emotions (e.g., excitement), whereas East Asian cultures preferred more balanced and low arousal emotional states (Tsai et al., 2006, 2007a; Vishkin and Tamir, 2023). Another study suggests that perceived cultural norms may explain cultural differences in reports of positive emotions (Oishi, 2002). Oishi’s (2002) investigation focused on self-reported emotional experiences among Asian Americans and European Americans. The study found variations in how the two groups reported past positive emotions, but no disparities in their real-time or current emotional experiences. That is, only when asked to reflect on their emotions retrospectively, European Americans reported higher positive emotions compared to Asian Americans, indicating that European Americans may perceive it as good to feel positive emotions and/or that they tend to show biases when asked to retrospectively judge their well-being.

The findings on the appropriateness of negative emotions compared to positive emotions are less conclusive: no or fewer cultural differences have been observed, as negative emotions are generally unpleasant and socially undesirable (Eid and Diener, 2001; Scollon et al., 2004, 2011; see also Wirtz et al., 2009).

Based on these research findings highlighting individualistic cultures’ emphasis on experiencing and expressing positive emotions (Eid and Diener, 2001; Tsai et al., 2006), we expect people with a European background compared to those with an East Asian background to think that it is appropriate to experience positive emotions in their culture. This perception of cultural norms of positive emotions is predicted to influence Europeans’ positive self-views, which, in turn, is expected to enhance their subjective well-being.

### The present study

Our study focuses on two aspects of subjective well-being: life satisfaction and domain satisfaction. While life satisfaction has been extensively examined, domain satisfaction—which explores satisfaction within specific life areas, such as family relationships—has received less attention in research (Diener et al., 1995, 2000). In our research, we address both life satisfaction and domain satisfaction, recognizing them as crucial, yet distinct, components of subjective well-being that are positively correlated and collectively contribute to our understanding of individuals’ overall subjective well-being.

Using a serial mediation model, the current study is the first to examine a series of psychological mechanisms for well-known cultural differences in subjective well-being. Specifically, we test whether cultural differences in subjective well-being (as indexed by both life satisfaction and domain satisfaction) are explained, in the first step, by cultural norms of positive emotions, and in the second, by cultural differences in positive illusions.

*H1*: Positive illusions are positively associated with higher life satisfaction and domain satisfaction.

*H2*: Europeans show more positive illusions compared to East Asians.

**Figure 1:**
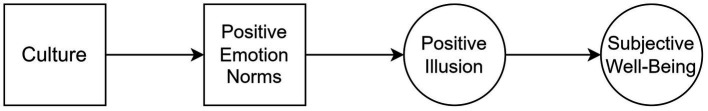
Conceptual serial mediation model. Hypothesis 3 tests serial mediation from culture to subjective well-being.

*H3*: Cultural differences in positive emotion norms contribute to cultural differences in positive illusions and life and domain satisfaction. (Figure 1).

## Methods

### Ethics statement

The study was approved by the Institutional Ethics Review Board at the university. Informed consent was obtained from all participants before they participated in the study.

### Procedure and participants

University students at a Canadian University participated in an online study for course credits. Participants were provided with a description of the study and were asked to complete the survey after providing their consent. The survey consisted of self-report measures, such as the subjective well-being and cultural norms of emotion questionnaire, and socio-demographic questions.

The convenience sample consisted of 296 university students. One hundred twenty individuals identified themselves as Caucasian/White (e.g., French, Austrian) and 136 individuals identified themselves as East Asian (e.g., Korean, Chinese). Out of these participants, 31 failed to answer the attention items correctly.^1^ Data from these participants were excluded from the statistical analyses, resulting in a final sample of 225 participants (112 Europeans and 113 East Asians). The mean age of the university student sample was 21.2 years (*SD* = 6.18 years) in the European sample and 20.7 years (3.29 years). Females accounted for 70% of the European sample and 69% of the East Asian sample.

### Measures

#### Measure of positive self-views

Positive self-views were modeled using two different measures: self-ratings of Big Five traits and self-ratings of performances and abilities.

#### Big five

Participants responded to the 44-item Big Five Inventory (BFI; John and Srivastava, 1999, Soto and John, 2009) using a 7-point Likert scale ranging from 1 (*strongly disagree*) to 7 (*strongly agree*). They were asked to think about their personality in general and provide their agreement to each statement starting with “I tend to.” The Big Five personality model has been widely used in psychological research and captures the broad individual differences in personality. Participants provided their agreement to the individual items representing one of the Big Five traits (Neuroticism, Extraversion, Openness, Agreeableness, Conscientiousness), and the items were averaged to generate scores for each of the Big Five traits (e.g., eight items for Extraversion).

Positive self-view was measured and modeled via ratings of the Big Five. Specifically, personality ratings were fitted to the structural equation model of the correlations between the Big Five traits (Anusic et al., 2009). Previous research has shown how this halo bias measure reflects individuals’ biases in self-ratings of personality characteristics. Halo bias separates a halo factor from the higher-order personality factors, which are true personality trait variances, and other sources of measurement error (see Anusic et al., 2009). The halo bias reflects individual’s positive bias of a single rater. Higher scores on the halo bias reflect a tendency to view oneself overly positively on evaluative traits (more extraverted, open, agreeable, emotionally stable, and conscientious). Importantly, we use halo bias (hereafter also referred to as *positive illusion*) to test the serial mediation hypothesis.

#### Bias measure

Another bias measure (hereafter referred to as the *bias measure*) was included to analyze the validity of the halo bias from the structural equation model of Big Five traits. The bias measure was assessed with participants’ ratings on distinct performances and abilities that are unrelated. Participants were asked how they would perform on each item on a 7-point scale ranging from 1 (*very bad*) to 7 (*very good*): performing on a scientific test of intelligence; performing in an athletic contest that requires you to jump as far as possible from standing; performing in a geography test that requires you to locate nations and name capitals; performing on a quiz with trivial pursuit questions; others rating my attractiveness based on a picture of my face. Higher ratings reflect positive self-views of one’s own abilities and characteristics (α = 0.71).

### Cultural norms for experiencing positive and negative emotions

The cultural norms for emotions were assessed by the following instructions: “In the following, we would like you to indicate how appropriate it is to experience and express certain emotions in everyday life. Treat each emotion and each situation separately. Do not consider them occurring in any particular order or to be connected with each other in any way.” Each item could be answered on a 7-point scale from 1 (*extremely inappropriate*) to 7 (*extremely appropriate*) on the following emotions: joy, affection, pride, contentment, anger, fear, sadness, guilt, excitement, happiness, contempt, surprise. Cultural norms regarding positive emotions (joy, affection, pride, contentment, excitement, happiness, surprise) and cultural norms regarding negative emotions (anger, fear, sadness, guilt, contempt) were separately calculated by averaging the items’ scores (both αs = 0.83).

### Life satisfaction

Satisfaction with life was measured with the first three items of the Satisfaction with Life Scale (Diener et al., 1985). The first three items have shown good reliability (“In most ways my life is close to my ideal,” “The conditions of my life are excellent,” and “I am satisfied with my life”) and have been validated among North Americans and Asians (Oishi, 2006). Participants provided their agreement to the statement on a 7-point Likert scale (*α* = 0.83).

### Domain satisfaction

Participants also provided their satisfaction with specific domains in life on a 7-point Likert scale (Schimmack et al., 2005) by providing their agreement to each statement starting with “I am satisfied with.” Four (out of six) items were averaged to reflect domain satisfaction (*α* = 0.71): my satisfaction with my parents, my academic performance, my healthy lifestyle, and my progress toward my goals in life. Items were selected based on their reliability and the correlations among them.

## Results

Analyses on the data were performed through confirmatory factor analysis (CFA) and structural equation modeling (SEM) using the R programming language (Version 4.3.1). The fit of the model was evaluated with the commonly used fit indices, including the comparative fit index (CFI), root mean square error of approximation (RMSEA), and standardized root mean square residual (SRMR). Values greater than 0.95 on the CFI and lower than.06 on the RMSEA indicate a good fit. The cutoff criteria for poor fit are CFI < 0.85, RMSEA >0.10, and SRMR >0.08 (Browne and Cudeck, 1993; Hu and Bentler, 1999). Overall, the findings supported the three hypotheses.

### The measurement model

Individuals’ tendencies to view themselves overly positively were measured via the halo bias factor (also referred to as positive illusion). First, personality items were averaged to represent each of the Big Five traits. Second, the halo model (Anusic et al., 2009) was fitted to self-ratings of the Big Five traits. The factor loading of the first factor was fixed to 1, and all other loadings were freely estimated. Third, additional significant correlations among the Big Five traits were included based on the theoretical model of higher order factors of personality and prior findings (Anusic et al., 2009). After examining post-hoc modification indices, the following traits were allowed to correlate with each other – agreeableness and conscientiousness, and agreeableness and emotional stability – reflecting the psychosocial maturity aspect of individuals’ personality and socialization processes. The modified model showed good fit, CFI = 0.99, RMSEA = 0.04, SRMR = 0.03.

Next, to test the convergent validity of the positive illusion factor, we analyzed the bias measure by fitting the factor to correlations between the five evaluative items using structural equation modeling. The first factor loading was fixed to 1. Post-hoc modification indices suggested that the single-factor model best fit the data when the residuals of two items (geography test item and trivial pursuit question item) were allowed to correlate with each other, CFI = 1.00, RMSEA = 0.01, SRMR = 0.03.

Next, to validate the current positive illusion measure, the bias measure was added to the measurement model of positive illusion/halo bias. The latent bias measure was correlated with the latent halo bias factor to examine convergent validity, CFI = 0.97, RMSEA = 0.04, SRMR = 0.06. The halo bias factor was positively correlated with the bias measure, providing support for the convergent validity of the positive illusion factor: *b* =0.30, 95% CI [0.17, 0.44]. These findings are consistent with prior findings that found convergent validity of the halo bias factor with other positive illusion factors (e.g., Anusic et al., 2009).

### Positive illusion and life satisfaction

In the next model, the latent life satisfaction factor (composed of three life satisfaction items) was added to the measurement model of the positive illusion model to examine the link between halo bias and life satisfaction. The factor loading of the first life satisfaction item on the latent life satisfaction factor was set to 1, and other loadings were freely estimated. The regression model with the positive illusion factor showed good fit, CFI = 0.99, RMSEA = 0.03, SRMR = 0.04. Consistent with Hypothesis 1, individuals who evaluated their personality more positively provided higher self-reported life satisfaction, *b* = 1.33, 95% CI [0.86, 1.81]. These findings suggest that people who have positive views of the self are also more likely to report that they are satisfied with their life in self-reports (Anusic et al., 2009; Kim et al., 2012, 2016).

### Positive illusion and domain satisfaction

The link between halo bias and subjective well-being was also investigated with domain satisfaction. Individuals who showed more positive illusions showed higher satisfaction with different domains in life, *b* = 0.71, 95% CI [0.35, 1.07], CFI = 0.95, RMSEA = 0.06, SRMR = 0.05.

### Culture, positive illusion, and subjective well-being

Finally, we added culture to the regression model. Culture (0 = Caucasian/White, 1 = East Asian) was added to the model and was assumed to predict differences in positive illusion, which then was assumed to predict life satisfaction (see Supplementary Table S1 for details). The original mediation path (Kim et al., 2012, 2016) via the halo bias factor was extended using domain satisfaction in the current research. In line with Hypothesis 2, European Canadians showed significantly more positive illusions compared to East Asians, *b* = 0.32, 95% CI [0.13, 0.52], in the model on life satisfaction; *b* = 0.41, 95% CI [0.23, 0.60], and in the model on domain satisfaction.

### Mediation path via emotion norms

Importantly, to understand the psychological mechanism of positive illusions, a psychological mediator was added: cultural norms of positive emotions (see Figure 1).

#### Positive emotions

**Figure 2:**
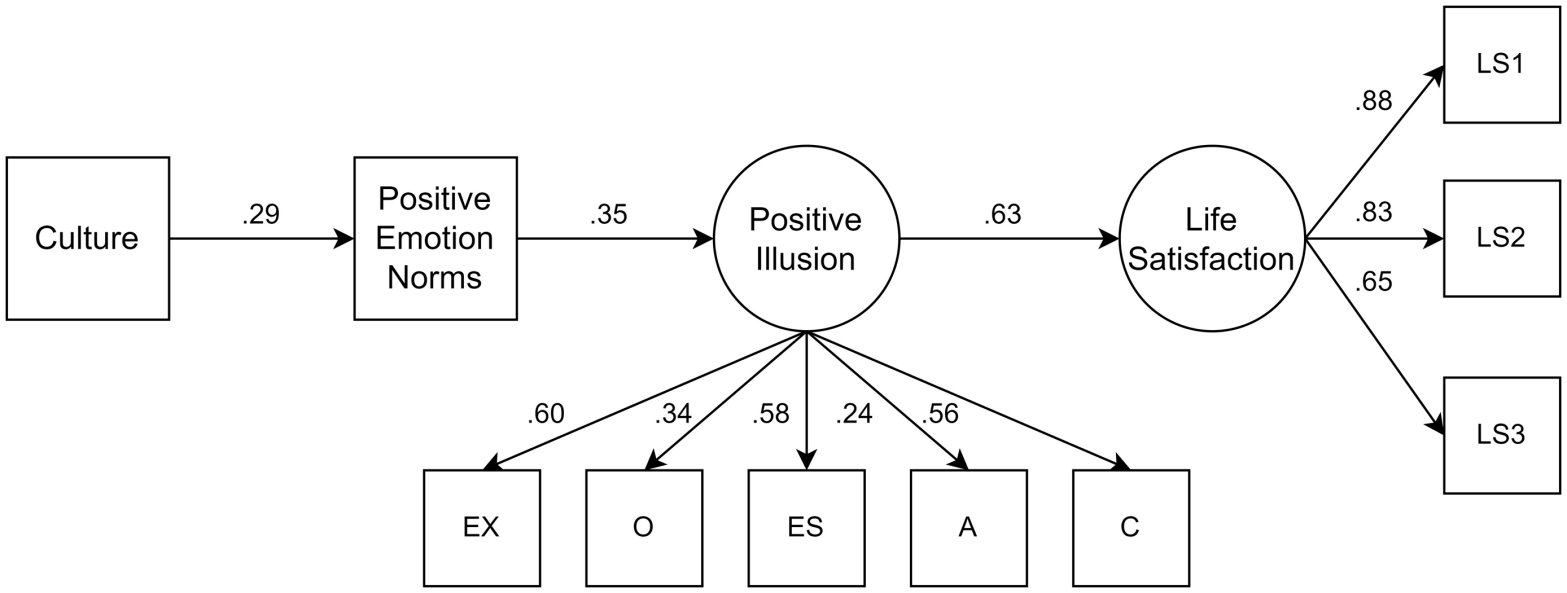
Serial mediation model with estimates. Numbers in the figure reflect standardized estimates. The correlations between the error variance of emotional stability and agreeableness (0.16), and agreeableness and conscientiousness (0.33), not shown in the figure, were significant. The figure illustrating the serial mediation model on domain satisfaction is available in the Supplementary material.

The mediation hypothesis via norms of positive emotions and positive illusions was tested. Consistent with Hypothesis 3, the results yielded a significant indirect effect of culture on life satisfaction via positive emotion norms, *d* = 0.16, 95% CI [0.06, 0.27], and domain satisfaction, *d* = 0.09, 95% CI [0.03, 0.16] via norms of positive emotions^3^ (see Figure 2; Table 1). Europeans were more likely to think that it is appropriate to experience and express positive emotion in daily life, which predicted their positive illusions, which in turn were associated with their higher life satisfaction and domain satisfaction (see Supplementary Figure S1 for the mediation model on domain satisfaction).

**Table 1:** Serial mediation path via norms of positive emotions and positive illusion.

Effect	β	Unstandardized estimates				*p*
		*B*	*SE*	*LL*	*UL*	
**Model – life satisfaction**						
Culture -> Positive emotion norms	0.287	0.416	0.093	0.234	0.597	0.00
Positive emotion norms -> Positive illusion	0.349	0.287	0.070	0.150	0.425	0.00
Positive illusion -> Life satisfaction	0.630	1.341	0.236	0.878	1.804	0.00
**Model – domain satisfaction**						
Culture -> Positive emotion norms	0.287	0.416	0.093	0.234	0.597	0.00
Positive emotion norms -> Positive illusion	0.380	0.246	0.063	0.123	0.370	0.00
Positive illusion -> Domain satisfaction	0.694	0.913	0.248	0.428	1.398	0.00

β, standardized regression coefficients; B, unstandardized regression coefficients; SE, standard error of the unstandardized regression coefficients; LL, lower limit of the 95% confidence interval; UL, upper limit.

Next, the mediation model with the direct path from culture to life satisfaction was examined. The indirect path through positive emotion norms and positive illusions was significant, *d =* 0.15, 95% CI [0.05, 0.26]. The total path from culture to life satisfaction was not significant, *d =* 0.25, 95% CI [−0.08, 0.58], and the direct path on life satisfaction was also not significant, *b* = 0.10, 95% CI [−0.22, 0.42].

The direct path was also tested in the model on domain satisfaction. The indirect path through positive emotion norms and positive illusions was significant, *d =* 0.08, 95% CI [0.02, 0.14], accounting for 25% of the total effect on domain satisfaction, *d* = 0.32, 95% CI [0.12, 0.52]. The direct path on domain satisfaction remained significant (*b* = 0.24, 95% CI [0.06, 0.42]).

We ran the indirect mediation model including individuals’ socioeconomic status as a covariate measured with the subjective socioeconomic status social ladder question.^4^ The indirect effects of culture remained significant after including the direct path from the social ladder factor to subjective well-being factors: life satisfaction, *d* = 0.15, 95% CI [0.05, 0.26]; domain satisfaction, *d* = 0.09, 95% CI [0.03, 0.16].^5^

#### Negative emotions

The results did not support mediation via negative emotions on subjective well-being: life satisfaction, *d* = 0.03, 95% CI [−0.02, 0.09]; domain satisfaction, *d* =0.04, 95% CI [−0.01, 0.08].

## Discussion

The present study offers a more complete picture of why people with a European background, compared to those with an East Asian background, show higher positive illusions and subjective well-being. That is, our research provides a complete model of culture, positive illusions, and subjective well-being by incorporating norms of positive emotions.

Here, we tested the possibility that cultural differences in norms of positive emotions explain why people with a European background, compared to those with an East Asian background, show positive illusions, which in turn is associated with their higher subjective well-being. The current research found that Europeans were more likely to think that it is appropriate to feel and express positive emotions, and these differences in cultural norms of positive emotions explained higher positive illusions and subjective well-being. The mediation pathways were supported after controlling for the socioeconomic status of individuals, which has been found to be a predictor of subjective well-being.

Previous studies have demonstrated that cultures differ in subjective well-being. In particular, cross-national and within-national studies have provided empirical evidence that people from individualistic cultures, compared to those from collectivistic cultures, show higher satisfaction with life (Veenhoven, 1991; Diener et al., 1995, 1999; Schimmack et al., 2005; Kim et al., 2012). One explanation for these cultural differences in subjective well-being appears to come from socio-economic factors. For instance, people from individualistic cultures, characterized by putting personal goals over collective goals, showed higher satisfaction with life due to their higher socio-economic status (Diener et al., 2010). Socio-economic affluence allows individuals to pursue activities and goals that align with their values, resulting in higher subjective well-being.

Recently, differences in positive self-views were suggested to explain cultural differences in subjective well-being. In several studies, people with a European background, compared to those with an Asian background, were found to show more positive biases in self-ratings of their personality (Kim et al., 2012, 2016). However, the psychological mechanism that may explain why Europeans show more positive illusions than Asians was not explored. The current findings on cultural norms of positive emotions provide important insights into the question of why some people, but not others, show more positive views of the self, as they think it is appropriate to feel and express positive emotions. The serial mediation results offer additional evidence underscoring the significance of accounting for distinct self-view patterns among cultural groups in research. Failing to consider these differences may cloud our comprehension of the cultural variations in subjective well-being and other psychological constructs.

### Cultural norms of positive emotions

Cultural research on norms of emotional experience and expression has found that people from individualistic cultures favor experiencing and expressing positive emotions (Diener et al., 2000; Eid and Diener, 2001; Tsai et al., 2006; Scollon et al., 2009, 2011). These differences can be attributed to several factors such as differences in values and beliefs, socialization, communication styles, historical factors, and societal factors (see Diener et al., 1995; Tsai et al., 2007b; Scollon et al., 2009). Individualistic cultures encourage open expression of positive emotions, whereas collectivistic cultures may put more emphasis on emotional restraint and humility than on the expression of positive emotions. The encouragement of humility in East Asian culture, rooted in Confucianism and Taoism, may foster the cultivation of modest thinking and the experience and expression of one’s thoughts and emotions among East Asians (Lee et al., 2001; Kim, 2023). The humility East Asians show is distinct from self-effacement and reflects the tendency of demonstrating humility in everyday life.

Many studies have found Europeans to show more positive emotions and a preference toward feeling and expressing positive emotions (Diener et al., 1995; Eid and Diener, 2001; Tsai et al., 2006, 2007b; Wirtz et al., 2009; Scollon et al., 2011). European Americans, compared to Asians, showed higher positive emotions in their retrospective reports (Diener et al., 2000; Oishi, 2002; Scollon et al., 2009; Wirtz et al., 2009). Furthermore, differences were observed depending on the arousal level of positive emotions. Tsai et al. (2007b) found that European Americans preferred high-arousal positive emotions (e.g., excitement), whereas Taiwanese Chinese preferred low-arousal positive emotions (e.g., contentment). In the current research, separate analyses were conducted to examine whether the indirect effect differed depending on the arousal level of positive emotions and types of emotions (i.e., self-conscious emotions). Self-conscious emotions, such as pride, have been associated with higher subjective well-being in the United States (vs. Japan), an individualistic culture, as these emotions are experienced in reference to the self and directly influences individuals’ self-esteem (Kitayama et al., 2000). Interestingly, in the current research, the influence of culture on subjective well-being, mediated by positive emotion norms and positive illusions, showed no variation across different types of positive emotions: people with a European background thought that experiencing positive emotions, whether low-arousal positive emotions or self-conscious positive emotions, was more appropriate compared to people with an East Asian background. These findings suggest that Europeans think that it is appropriate to feel positive emotions in general rather than specific types of positive emotions.

Past findings regarding negative emotions are inconsistent. Some research has found cultural differences in the appropriateness of negative emotions, whereas others have not (Eid and Diener, 2001; Scollon et al., 2011). In the present study, we tested the mediation pathway via cultural norms of negative emotions and did not find empirical evidence to support a mediation path via negative emotions. These findings align with prior findings on the focus on the positives in individualistic cultures, finding North Americans, compared to Asians, put more emphasis on their children’s successes and praise them more (Oishi and Sullivan, 2005; Uchida and Kitayama, 2009).

Further work needs to be carried out to investigate the psychological mechanism of positive illusions and subjective well-being in nationally representative samples of various countries that vary in their cultural values and socio-ecological factors to generalize the current findings. For example, the historical routes and societal factors differ among collectivistic cultures. South Korea is similar in terms of its cultural orientation to Singapore; however, the two cultures differ in their cultural history, socioeconomic status, and societal factors, including the ethnic makeup of the population and GDP. Additionally, it would be interesting to examine other psychological factors that may explain why people from individualistic cultures versus other cultures show more positive illusions of the self.

## Data availability statement

The datasets presented in this study can be found in online repositories. The names of the repository/repositories and accession number(s) can be found at: https://osf.io/7nv5q/?view_only=dd2db904da264d74bdd3877593b181b9.

## Ethics statement

The studies involving humans were approved by Veronica Jamnik, Chair, Human Participants Review Committee, York University. The studies were conducted in accordance with the local legislation and institutional requirements. The participants provided their written informed consent to participate in this study.

## Author contributions

HK: Writing – original draft, Writing – review & editing, Conceptualization, Investigation, Data Curation, Methodology, Formal analysis, Visualization. JS: Writing – original draft, Writing – review & editing.

## Footnotes

^1^For power of 0.95 at a = 0.05 (one-sided), the estimated sample size per group was approximately 88 participants per group. The data, analysis code, and other supplementary materials are available at: https://osf.io/7nv5q/?view_only=dd2db904da264d74bdd3877593b181b9. The study was not preregistered. Data collection started in May 2019 and ended in May 2020.

^3^The fit of the model was marginal for life satisfaction, CFI = 0.87, RMSEA = 0.10, SRMR = 0.08; for domain satisfaction, CFI = 0.84, RMSEA = 0.09, SRMR = 0.08. The CFI of the initial model on domain satisfaction was low, although the RMSEA and SRMR values were acceptable. After examining post hoc modification indices, one correlated error among the Big Five traits was added to the model. This modification improved the CFI to 0.84, which is below the cutoff value. The criteria are used as a rule of thumb and not a strict requirement. The modified domain satisfaction model did not significantly change the regression coefficients and direct effects in the regression models (e.g., the link between positive illusion and domain satisfaction).

^4^An individual’s socio-economic status was measured using the MacArthur Scale of Subjective Social Status (Adler et al., 2000), a single-item measure depicting subjective social status as a 10-rung ladder. Participants ranked themselves on this ladder relative to others in society, with the top rung (10) representing those who are best off and the bottom rung (1) representing those who are worst off.

^5^Mediation models including either low arousal positive emotion or high arousal positive emotions were analyzed. The indirect effects of culture on life satisfaction were significant: low arousal positive emotion (contentment), *d* = 0.12, 95% CI [0.03, 0.21]; and high arousal positive emotions (joy, excitement, surprise), *d* = 0.12, 95% CI [0.03, 0.20]. The indirect effects on domain satisfaction were also significant: low arousal positive emotion, *d* = 0.07, 95% CI [0.01, 0.13]; and high arousal positive emotions, *d* = 0.08, 95% CI [0.02, 0.13]. Additionally, a mediation model with the positive self-conscious emotion (pride) was examined. The indirect effect via pride was small but significant: *d* = 0.09, 95% CI [0.01, 0.17] for life satisfaction and *d* = 0.05, 95% CI [0.00, 0.10] for domain satisfaction.
