# Supplementary material for: Toward an explanation of cultural differences in subjective well-being: the role of positive emotion norms and positive illusions
Source: Front Psychol. 2024 Jun 20;15:1356172. doi: 10.3389/fpsyg.2024.1356172 (PMC11222831; doi:10.3389/fpsyg.2024.1356172)
Supplement: Supplementary file 1 [file Table_1.docx]

**Supplemental Online Material**

**Table S1**

*Mediation Path via Positive Illusion*

| Effect | β | Unstandardized Estimates | | | | *p* |
| --- | --- | --- | --- | --- | --- | --- |
|  |  | *B* | *SE* | *LL* | *UL* |  |
| **Model – life satisfaction** |  |  |  |  |  |  |
| Culture →  Positive illusion | .277 | .324 | .098 | .132 | .517 | .001 |
| Positive illusion →  Life satisfaction | .617 | 1.335 | .241 | .862 | 1.808 | .00 |
| **Model – domain satisfaction** |  |  |  |  |  |  |
| Culture →  Positive emotion norms | .420 | .412 | .093 | .230 | .595 | .00 |
| Positive illusion →  Domain satisfaction | .700 | .872 | .211 | .431 | 1.313 | .00 |

*Note.* β = standardized regression coefficients; *B* = unstandardized regression coefficients; *SE* = standard error of the unstandardized regression coefficients; *LL* = lower limit of the 95% confidence interval; *UL* = upper limit.

**Figure S1**

*Serial Mediation Model on Domain Satisfaction*


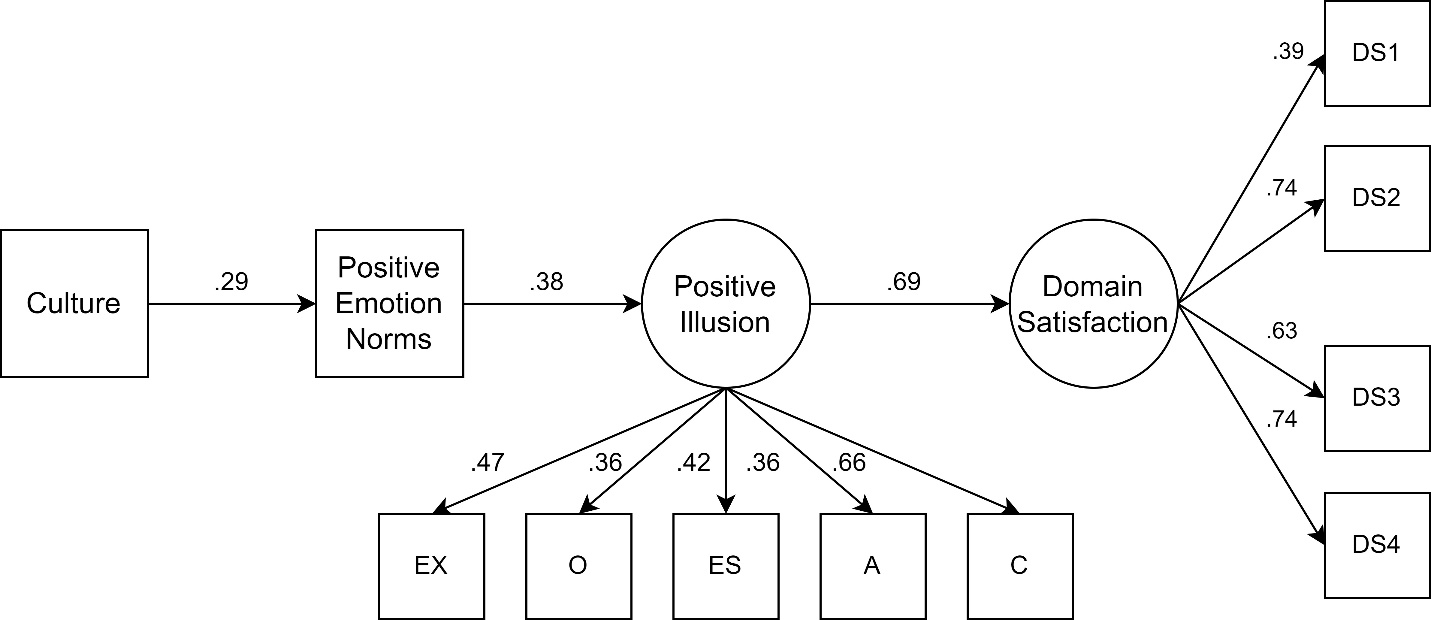


*Note*. All displayed coefficients are standardized regression coefficients and significant at *p* < .05. Additionally, three correlated errors among the Big Five traits, not shown in the figure, were significant: the correlation between agreeableness and conscientiousness (*r* = .22), between emotional stability and agreeableness (*r* = .15), and between emotional stability and extraversion (*r* = .26).

After examining *post hoc* modification indices in the mediation model, an additional correlated error among the Big Five traits—specifically between emotional stability and extraversion—was included in the model, along with the two previously added correlated errors among the Big Five traits. Although including this correlation improved model fit, the regression results remained the same with and without it.

The association between extraversion and emotional stability has been observed in prior research. This correlation has been attributed to their shared conceptual basis, particularly regarding emotional aspects, as well as their common biological foundation.
